# The effects of dual PPAR*α*/*γ* agonism compared with ACE inhibition in the BTBRob/ob mouse model of diabetes and diabetic nephropathy

**DOI:** 10.14814/phy2.13186

**Published:** 2017-03-14

**Authors:** Anette Ericsson, Pernilla Tonelius, Mark Lal, Alan Sabirsh, Gerhard Böttcher, Lena William‐Olsson, Maria Strömstedt, Camilla Johansson, Gina Hyberg, Sofia Tapani, Ann‐Cathrine Jönsson‐Rylander, Robert Unwin

**Affiliations:** ^1^ Cardiovascular & Metabolic Disease Innovative MedicinesAstraZeneca R&D GothenburgMölndalSweden; ^2^ Drug, Safety & MetabolismAstraZeneca R&D GothenburgMölndalSweden; ^3^ Discovery Sciences Innovative Medicines Research UnitAstraZeneca R&D GothenburgMölndalSweden

**Keywords:** angiotensin‐converting enzyme, BTBRob/ob mouse, diabetic kidney disease, peroxisome proliferator‐activated receptor

## Abstract

The leptin‐deficient BTBRob/ob mouse develops progressive albuminuria and morphological lesions similar to human diabetic nephropathy (DN), although whether glomerular hyperfiltration, a recognized feature of early DN that may contribute to renal injury, also occurs in this model is not known. Leptin replacement has been shown to reverse the signs of renal injury in this model, but in contrast, the expected renoprotection by angiotensin‐converting enzyme (ACE) inhibition in BTBRob/ob mice seems to be limited. Therefore, to investigate the potential renal benefits of improved metabolic control in this model, we studied the effect of treatment with the dual peroxisome proliferator‐activated receptor (PPAR) *α*/*γ* agonist AZD6610 and compared it with the ACE inhibitor enalapril. AZD6610 lowered plasma glucose and triglyceride concentrations and increased liver size, but had no significant effect in reducing albuminuria, whereas enalapril did have an effect. Nephrin and WT1 mRNA expression decreased in the kidneys of BTBRob/ob mice, consistent with podocyte injury and loss, but was unaffected by either drug treatment: at the protein level, both nephrin and WT1‐positive cells per glomerulus were decreased. Mesangial matrix expansion was reduced in AZD6610‐treated mice. GFR, measured by creatinine clearance, was increased in BTBRob/ob mice, but unaffected by either treatment. Unexpectedly, enalapril‐treated mice showed intrarenal arteriolar vascular remodeling with concentric thickening of vessel walls. In summary, we found that the BTBRob/ob mouse model shows some similarities to the early changes seen in human DN, but that ACE inhibition or PPAR *α*/*γ* agonism afforded limited or no kidney protection.

## Introduction

The global “epidemic” of type 2 diabetes will lead to an increase in the number of patients developing chronic kidney disease (CKD) and end‐stage renal disease (ESRD) eventually requiring dialysis and renal transplantation. The typical early renal structural lesions seen in diabetes include thickening of the glomerular basement membrane (GBM) and mesangial expansion, with later more advanced glomerular scarring and classical Kimmelstiel–Wilson nodule formation; podocytes, the tubulointerstitium, and arterioles are also affected with podocyte loss, interstitial fibrosis, and arteriolar hyalinosis (Fioretto and Mauer 2007).

To improve our understanding of the pathogenesis of diabetic nephropathy (DN), and to identify potential therapeutic targets, better animal models of human DN are still needed. The BTBRob/ob mouse is one model that has been reported to mimic reliably many of the typical early pathological features of human DN. These mice have been shown to develop progressive albuminuria with early accumulation of mesangial matrix and loss of glomerular podocytes, and in older mice there is GBM thickening, diffuse mesangial sclerosis, arteriolar hyalinosis, and mesangiolysis (Hudkins et al. 2010).

Peroxisome proliferator–activated receptors (PPARs) play an important role in metabolic diseases associated with hyperlipidemia and insulin resistance, and in diabetes mellitus. Both PPAR*α* and ‐*γ* are expressed in the kidney (Yang et al. 1999; Guan and Breyer 2001), and their agonists have shown renoprotective effects in type 2 diabetes. Although PPAR*α* and ‐*γ* agonists have shown some promise as treatments for DN (Keech et al. 2005; Sarafidis et al. 2010), their adverse side effects can include fluid retention, increased risk of myocardial infarction (Nesto et al. 2003), and elevated serum creatinine levels (McQuade et al. 2008); although the latter is often short‐lived and reversible (Mychaleckyj et al. 2012).

The renoprotective effects of PPAR agonists have been demonstrated in the leptin receptor‐deficient and insulin‐resistant db/db mouse model of DN. Fenofibrate (PPAR*α* agonist) treatment in this model was shown to reduce glucose and insulin levels, albuminuria, glomerular hypertrophy, and mesangial expansion (Park et al. 2006). In the same mouse model, rosiglitazone (PPAR*γ* agonist) was found to lower blood glucose and triglycerides levels, but was without effect on insulin levels, and failed to decrease albuminuria and mesangial expansion (Chodavarapu et al. 2013). The dual PPAR*α*/*γ* agonist, tesaglitazar, has also been shown to decrease blood glucose, insulin, and triglyceride concentrations, as well as albuminuria and glomerular lesions (Cha et al. 2007).

In a recent study using the PPAR*α* agonist CP‐900691 in the BTBRob/ob mouse model, Askari et al. (2014) reported that while treatment improved glucose and triglyceride levels, it did not affect insulin levels, albuminuria, or mesangial expansion. In contrast, the widely used ACE inhibitor enalapril, commonly prescribed to patients with DN, has been shown to reduce albuminuria in the BTBRob/ob mouse model, but with only modest effects on renal histology (Pichaiwong et al. 2013).

Thus, given the conflicting data discussed above and the uncertainty over the beneficial renal effects of PPAR agonists in different mouse models, as well as the limited studies of renoprotection particularly in the BTBRob/ob model of DN, we wanted to investigate the effect of a dual PPAR*α*/*γ* agonist, AZD6610, and compare it with RAS blockade using the ACE inhibitor enalapril, starting treatment at 17 weeks of age when the mice consistently show features of DN. Our aim was to evaluate the efficacy of interventional treatment in mice consistently showing features of established DN, rather than as preventive therapy, starting at the onset of albuminuria (~6–8 weeks age) with only modest morphological alterations, so as to better reflect current treatment strategies in patients.

## Materials and Methods

### Ethical approval

Experimental procedures were approved (ethical application number 109‐2012) by the Regional Laboratory Animal Ethics Committee of Gothenburg, Sweden. All procedures conform to the Swedish Animal Welfare Act and regulations SJVFS 2012: 26.

### Animals

BTBR.V(B6)‐Lepob/WiscJ stock no. 004824 mice were purchased from Jackson Laboratories (Bar Harbor, ME). Mice were delivered to our facility at ages 4–6 weeks. Animals were housed in communal cages (2–3 mice/cage) equipped with heated areas. The bedding was changed twice weekly and mice had free access to chow diet. The mice were randomized according to body weight, fasting glucose, and HbA_1C_ into the following groups at 8 weeks of age: (1) BTBRob/ob controls (*n* = 12); (2) BTBRob/ob treated with enalapril (Sigma E6888‐5G) dissolved in drinking water 200 mg/l (40 mg/kg/day) with a noncaloric sweetener (Splenda, 240 mg/l) from 14 weeks of age (*n* = 7); (3) BTBRob/ob treated with the dual PPAR*α*/*γ* agonist AZD6610 added to the diet at the glucose lowering dose of 0.012 mg/g (3 *μ*mol/kg/day) started at 17 weeks of age, which was preceded by a nonglucose lowering dose (1 *μ*mol/kg/day) started at 14 weeks of age (*n* = 8); (4) BTBR lean control mice (*n* = 5). The main aim of the study was to evaluate the efficacy of the treatments in mice with established DN (at 14–17 weeks age), rather than as a preventive therapy. Only female mice were used in these experiments.

At the age of 24 weeks, all mice were anesthetized with isoflurane and loss of pedal reflexes confirmed before the mice were euthanized by exsanguination and disruption of blood flow. Another separate group of male BTBRob/ob (*n* = 5) mice that had similar blood chemistry to females (data not shown) and a group of lean control BTBR mice (*n* = 5) were used to measure glomerular filtration rate (GFR) more accurately than by creatinine clearance using FITC‐sinistrin clearance. Other separate subgroups of female BTBRob/ob (*n* = 6) and lean (*n* = 6) mice were used to measure systolic blood pressure in conscious mice by the tail cuff method.

### Blood analyses

Repeated blood glucose and HbA_1C_ levels were measured in the 3‐h fasted awake mouse using Accu‐Chek (Roche, REF 05599415370) and HbA_1C_ Now+ (Bayer, REF 81611409‐3038), respectively. Creatinine was analyzed on a LC‐MS/MS system consisting of a Waters Quattro Premiere mass spectrometer and an Agilent 1100 HPLC. Column was a Phenomenex Kinetex HILIC 2.6 *μ*, 2.1 × 100 mm. Mobile phase A was acetonitrile and mobile phase B was 40 mmol/L ammonium formate. Gradient was from 5% B to 50% B in 10 min with 5 min equilibration time. As internal standard, 0.2 *μ*mol/L d_3_‐creatinine in ACN:H_2_O, 9:1 was used. On termination at 24 weeks of age, blood samples were collected and separated plasma were analyzed for insulin and adiponectin using radioimmunoassays (Cat No. RI‐13K and MADP‐60HK, Merck Millipore, Darmstadt, Germany) and triglycerides using an enzymatic colorimetric method (Kit No. 12146029 triglycerides/GB, Roche Diagnostics GmbH, Mannheim, Germany).

### Urine analyses

To determine albumin excretion rates (AER), 13‐h urine collections were obtained from mice housed in metabolic cages. Urine albumin concentration was measured using a mouse‐specific albumin ELISA (E‐90AL, ICL). Urine creatinine was measured by the Jaffé method (Creatinine FS, REF117119910021, DiaSys). Estimated glomerular filtration rate as measured by creatinine clearance (CCr, mL/min) was calculated as: (Urine Cre × Urine volume [mL])/(Plasma Cre × Time [min]).

### GFR by FITC‐sinistrin clearance

To assess GFR in the BTBRob/ob mouse model, we used the two‐compartment model of two‐phase exponential decay as described previously for FITC‐inulin in conscious mice (Qi et al. 2004). However, in this experiment, we used a single bolus injection of 0.1 mL of 15 mg/mL FITC‐sinistrin (Mannheim Pharma & Diagnostics GmbH, Mannheim, Germany) that was given intravenously to male BTBRob/ob and lean control mice. Repeated tail vein blood sampling was carried out at 3, 7, 10, 15, 35, 55, and 75 min postinjection. FITC‐sinistrin concentration was measured using a Beckman Coulter Paradigm Detection System (Li et al. 2012).

### Histopathology

Tissue samples were fixed in buffered formaldehyde solution, dehydrated, and embedded in paraffin wax. Two *μ*m sections were cut and stained with hematoxylin–eosin, periodic‐acid–shiffs reagent (PAS), or with immunohistochemistry reagents as described below. Histopathological assessment of mesangial matrix accumulation (PAS), podocyte markers (nephrin and WT1), and arteriolar wall thickening (*α*‐SMA) was performed, and the sections were scored semiquantitatively on a scale of 0 (*normal*) to 5 (*severe*).

Glomerular basal membrane (GBM) thickness was assessed using electron microscopy (Jeol 1400 Transmission Electron Microscopy) over three glomeruli per kidney, images (*n* = 10–14) taken at 12,000× magnification and mean basal membrane thickness (nm) calculated.

### Immunohistochemistry

Immunohistochemistry staining was carried out for the podocyte markers nephrin and WT1 (see Table 1 for antibody details).

**Table 1:** Primary antibody details

Primary antibody	Host species	Dilution	Supplier
Nephrin	Guinea pig	1:1000	Acris
WT1	Rabbit	1:200	Calbiochem
*α*‐SMA	Mouse	1:100	Dako

*α*‐SMA, *α*‐smooth muscle actin.

A novel method was introduced to allow automated unbiased quantification of nephrin and WT1 staining in multiple, adjacent, immunostained, 2‐*μ*m transverse kidney sections that were digitized using slide scanners (Zeiss Axioscan or Zeiss Mirax, Carl Zeiss AG, Munich, Germany), fitted with a 20× air objective yielding images with 4.545 pixels/*μ*m. The resulting virtual slides were imported into Visiopharm Integrator System software (version 5.3, Visiopharm, Hørsholm, Denmark).

For analysis, the tissues from sequential sections were aligned and then resampled at 1.138 pixels/*μ*m using systematic random sampling of the cortical region with coverage of 60%. Machine learning and Bayesian classification (Visiopharm, Visiomorph DP module), followed by morphological filtering, identified tissues and stained areas. Individual glomeruli were identified using nephrin staining and then used to generate regions of interest on the adjacent tissue section that had been stained for WT1 expression. For each glomerulus, the number of WT1‐positive nuclei and the fractional area of WT1 staining were calculated.

### Real‐time quantitative PCR

RNA was extracted from kidney cortex and reverse transcribed into cDNA. TaqMan gene expression assays specific for nephrin (*Nphs1*, Mm00497828_m1), *WT1* (Mm01337048_m1), *Tnf* (Mm00443258_m1), *Ccl2* (Mm00441242_m1), *CD68* (Mm03047340_m1), *Gapdh* (Mm99999915_g1), and *Hprt* (Mm01324427_m1) (Applied Biosystems, Waltham, MA) were used. Relative quantification was obtained using the ΔΔCt method using Gapdh and Hprt for normalization.

### Study design

The objective of the study was to investigate the efficacy of dual PPAR*α*/*γ* agonist versus ACE inhibition in BTBRob/ob mice starting treatment at the age of 14–17 weeks. An a priori power analysis of historical in‐house data of albuminuria was performed to determine sample sizes. For a power of *β *= 80% at a significance level of *α *= 5%, a treatment effect from baseline albuminuria of 45–55% is detectable with *n* = 5–12 in each group. Where the upper *n*‐number is selected for the control group due to multiple comparisons in Dunnett's post hoc tests.

### Statistical analysis

**Figure 1:**
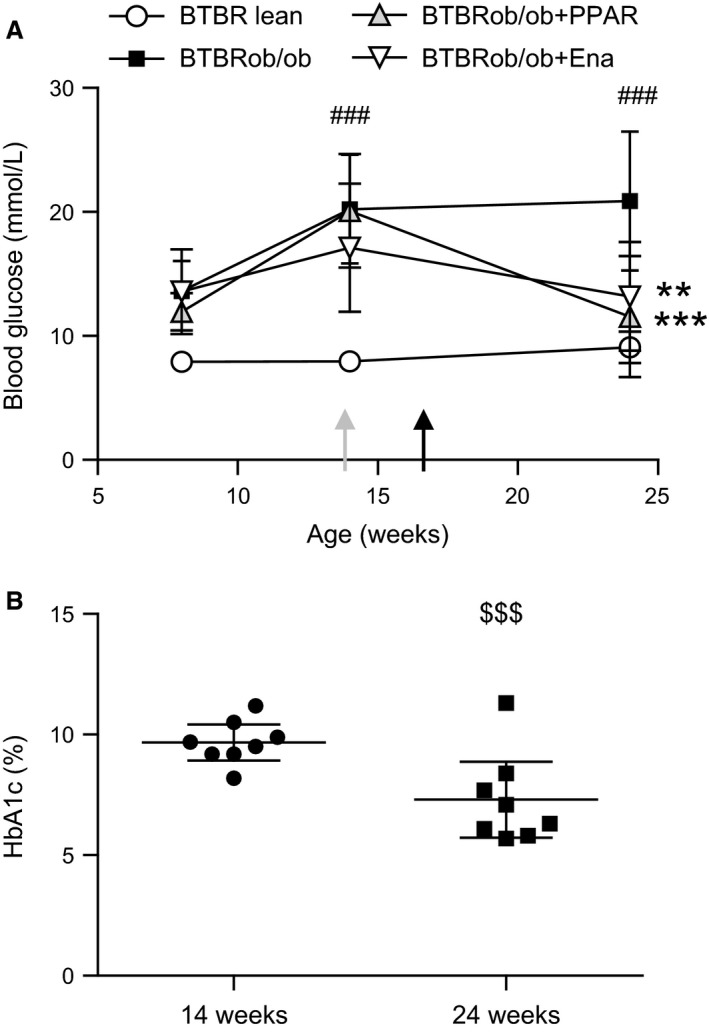
Blood glucose levels before and after treatment with a PPAR *α*/*γ* agonist or ACE inhibitor. (A) Blood glucose levels in conscious 3‐h fasted BTBRob/ob female mice (*n* = 12) measured at 8 and 14 weeks of age before treatment with enalapril (*n* = 7) or PPAR *α*/*γ* agonist (AZD6610, *n* = 8) and after treatment at 24 weeks age. The time point of starting the treatments are shown with arrows (gray, enalapril; black, PPAR). Repeated measures two‐way ANOVA and Dunnett's tests were used. (B) HbA_1C_ measured in 3‐h fasted BTBRob/ob mice before (14 weeks, *n* = 8) and after PPAR treatment (24 weeks, *n* = 8). Statistical analysis was done using a paired *t*‐test. Values are shown as means with 95% confidence intervals. *P < 0.05 compared with untreated BTBRob/ob and ^#^ *P* < 0.05, ^##^ *P* < 0.01 compared with BTBR lean. ^$$$^ *P* < 0.001 compared to before start of PPAR treatment. Ena, enalapril.

One‐way analysis of variance (ANOVA) followed by Dunnett's two‐sided post hoc tests for multiple comparisons were used to analyze data on blood and weight parameters, GBM thickness, and on mRNA expression data. Urine data, including creatinine clearance (CCr), albumin excretion rate (AER), and albumin creatinine ratio (ACR) show a right skewed distribution and so data were log‐transformed prior to performing ANOVA. Due to the small sample size, regular tests of normality have low power and were not performed. However, visual inspection of diagnostic plots after log transformation indicated a better fit to a log‐normal distribution. The same transformation was used for the quantitative computer‐generated histological evaluation; all data were normalized to the geometric mean value for the lean control group. Data were log‐transformed prior to using standard parametric tests as described above. Repeated measures on the same subject (Figs. 1A and 4) were analyzed with a repeated measures two‐way ANOVA and following appropriate Dunnett's two‐sided post hoc tests for correction of multiple comparisons. If *P* < 0.05 or if 95% confidence intervals were not overlapping, results were considered significantly different. Statistical analyses were performed with Graphpad^®^ 5.0 for Windows^®^.

## Results

### Characteristics of PPAR*α*/*γ* ligand‐ and enalapril‐treated BTBRob/ob mice compared with controls

All BTBRob/ob mouse groups had higher body weight compared with age‐matched lean BTBR controls; body weight was unaffected by either treatment (Table 2). Liver weights of BTBRob/ob mice were twofold higher than lean controls, reflecting obesity‐induced hepatic steatosis and increased triglyceride content, and a mean difference of 6.1 ± 1.8 g triglycerides/100 g liver (90% confidence interval 2.9 to 9.3), which is a recognized feature of the ob/ob mutation. Hepatomegaly is also a known effect of PPAR*α* agonism in rodents and in the present study a further increase (71%) in liver weight in AZD6610‐treated BTBRob/ob mice was also observed. Increased kidney weight was found in all BTBRob/ob mice without any visible effect of treatment. Furthermore, in a small subgroup of female mice, blood pressure was not statistically different between BTBRob/ob (93.8 ± 2.2 mm Hg) and BTBR lean controls (102.6 ± 3.0 mmHg). Blood pressure was not measured in the treatment groups.

**Table 2:** Weights and blood chemistry at 24 weeks age in female mice

	BTBR lean	BTBRob/ob	BTBRob/ob + PPAR	BTBRob/ob + Ena
Body weight (g)	34.5 (3.1)	71.6 (6.1)###	70.5 (10.7)	71.9 (7.6)
Liver weight (g)	1.7 (0.1)	3.8 (0.8)###	6.5 (1.5)**	3.8 (0.9)
Total kidney weight (g)	0.39 (0.02)	0.61 (0.13)###	0.63 (0.06)	0.59 (0.06)
HbA1C (%)	4.8 (0.2)	10.5 (2.4)###	7.3 (1.9)**	7.8 (2.2)*
Insulin (nmol/L)	0.3 (0.0)	2.3 (2.6)	3.4 (3.2)	6.3 (5.4)
TG (mmol/L)	0.5 (0.1)	2.2 (1.0)###	1.0 (0.2)**	1.8 (0.6)
Adiponectin (nmol/L)	672 (271)	278 (116)###	226 (95)	312 (175)

Data are presented as means and standard deviations for the groups: BTBR lean (*n* = 5), BTBRob/ob (*n* = 11–12), BTBRob/ob + PPAR (*n* = 7–8), and BTBRob/ob + Ena (*n* = 7). ^###^ *P* < 0.001 versus BTBR lean and *P < 0.05, **P < 0.01 versus BTBRob/ob vehicle control using ANOVA and Dunnett's post hoc test. Ena, enalapril; HbA1C, glycated hemoglobin A1C; TG, triglycerides.

Terminal blood samples were taken from the anesthetized 3‐h fasted mouse for insulin and TG analyses, whereas glucose and HbA_1C_ was measured in blood samples from awake 3‐h fasted mice (Table 2 and Fig. 1). The BTBRob/ob mouse was hyperglycemic (confirmed by elevated HbA_1C_ levels) and dyslipidemic, with high plasma triglyceride concentrations. Treatment with the PPAR*α*/*γ* agonist reduced plasma lipid concentrations, but we were unable to detect any change in insulin levels. The glucose‐lowering effect of AZD6610 occurred within 5 days of starting treatment and was readily evident from a marked decrease in daily water intake (pretreatment 16.1 [8.7] vs. posttreatment 9.2 [4.9] mL water/day) that was sustained throughout the study, and reduced urinary glucose excretion confirmed by urine dipstix testing (data not shown). Blood glucose was measured in 3‐h fasted conscious mice at different time points (Fig. 1A). The BTBRob/ob mice had elevated blood glucose levels compared with lean controls, and following 7 weeks of PPAR*α*/*γ* agonist treatment (age 17–24 weeks), blood glucose (Fig. 1A) and HbA_1C_ (Fig. 1B) were reduced. There was one mouse that did not respond to AZD6610 treatment and had persistently high plasma glucose (25.4 mmol/L) and triglyceride (1.4 mmol/L) concentrations compared with the other AZD6610‐treated mice (7.4–12.5 mmol/L glucose and 0.7–1.0 mmol/L triglycerides). At 24 weeks of age, plasma glucose and HbA_1C_ levels in the enalapril treatment group did reach a statistically significant difference compared with the BTBRob/ob control group. The hormone adiponectin was measured in terminal plasma to assess insulin sensitivity. Adiponectin levels were decreased in the BTBRob/ob mice compared with lean control mice; however, because of the large variability in the small sample size available for assay, we could not detect any effect of either treatment.

### Renal function

**Figure 2:**
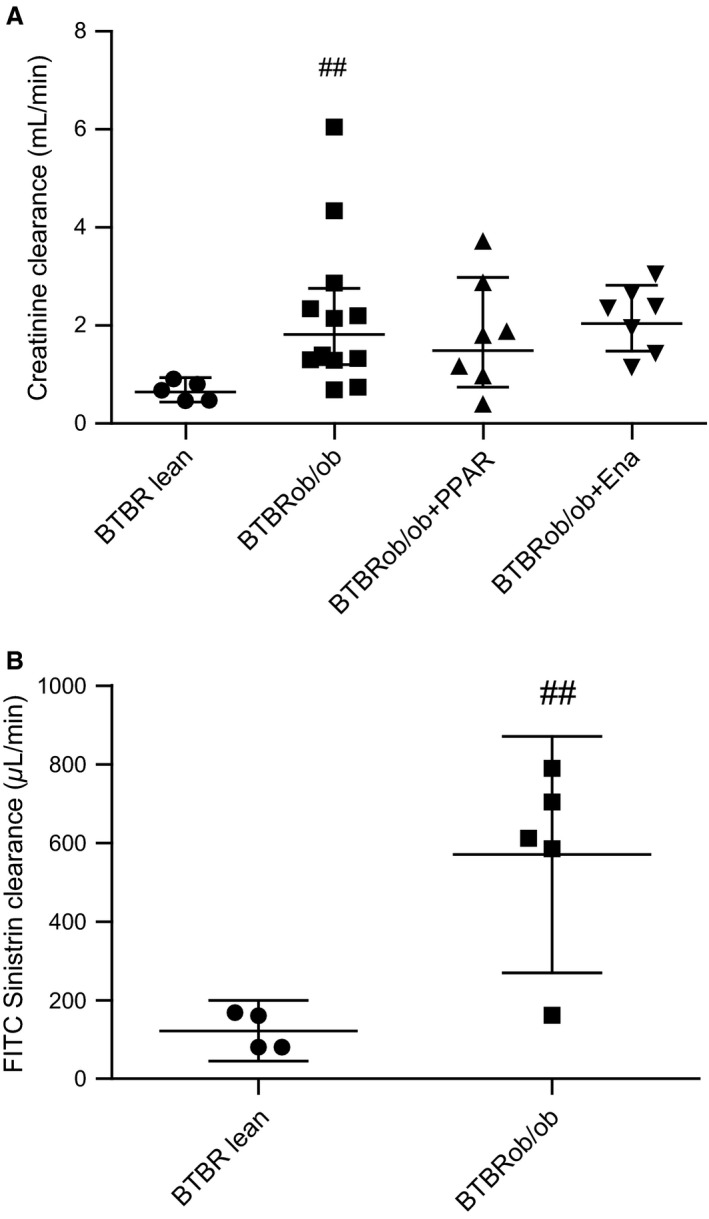
Glomerular filtration rate measured by creatinine clearance or FITC‐sinistrin clearance indicates hyperfiltration is present at 24 weeks of age. Creatinine clearance (A) was measured by use of metabolic cages for 13 h in all female mouse groups at 24 weeks age (BTBR lean, *n* = 5; BTBRob/ob, *n* = 12; BTBRob/ob + PPAR,* n* = 7; BTBRob/ob + Ena, *n* = 7). Values are shown as geometric means with 95% confidence intervals and one‐way ANOVA followed by Dunnett's multiple comparisons test were used on log‐transformed data. FITC‐sinistrin clearance (B) was measured by single bolus FITC‐sinistrin injection and repeated blood sampling in a subset of age‐matched male mice (BTBR lean, *n* = 4; BTBRob/ob, *n* = 5) and data were analyzed statistically using Student's *t*‐test. Values are shown as means with 95% confidence intervals. ^##^ *P* < 0.01 compared with BTBR lean. Ena, enalapril.

**Figure 3:**
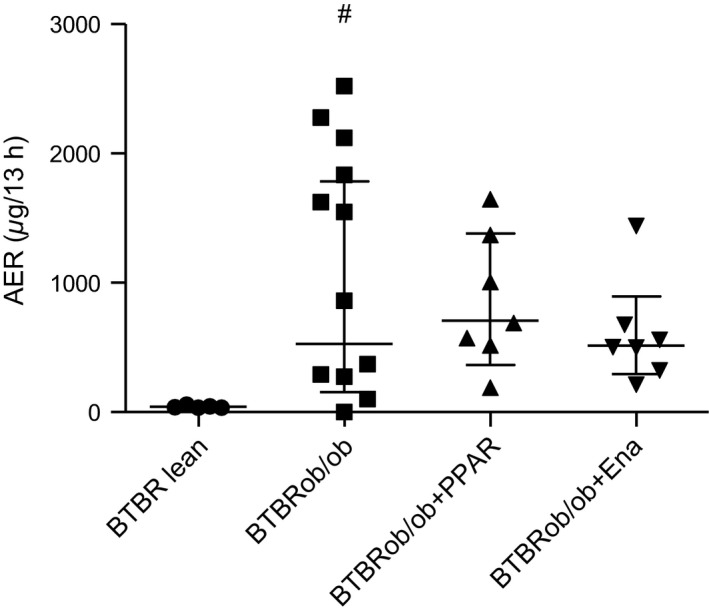
Albumin excretion rate is increased in the BTBRob/ob mouse. Albumin excretion rate (AER) was measured by use of metabolic cages for 13 h in all female mouse groups at 24 weeks age (BTBR lean, *n* = 5; BTBRob/ob, *n* = 12; BTBRob/ob + PPAR,* n* = 7; BTBRob/ob + Ena, *n* = 7). One‐way ANOVA followed by Dunnett's multiple comparisons test were used on log‐transformed data. Values are shown as geometric means with 95% confidence intervals. ^#^ *P* < 0.01 compared with BTBR lean. Ena, enalapril.

**Figure 4:**
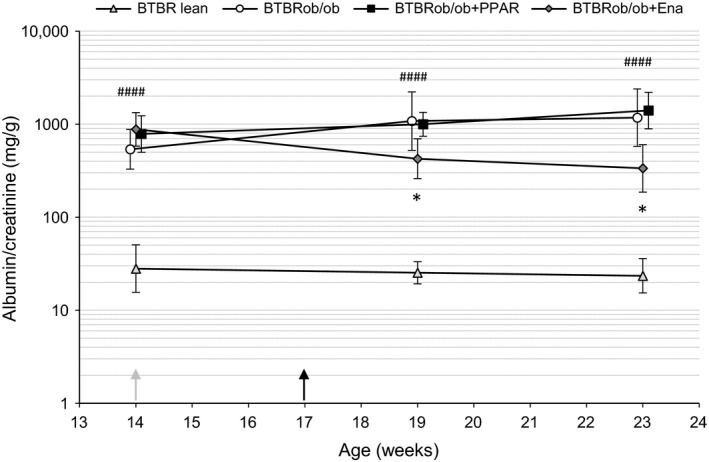
ACE inhibition reduces spot urine albuminuria, but PPAR *α*/*γ* agonism has no effect. Albuminuria measured in female mice as albumin:creatinine ratio (ACR) in spot urine (BTBR lean, *n* = 5; BTBRob/ob, *n* = 12; BTBRob/ob + PPAR,* n* = 8; BTBRob/ob + Ena, *n* = 7). The time points of starting the treatments are shown with arrows (gray, enalapril; black, PPAR). Repeated measures two‐way ANOVA and Dunnett's tests were used on log‐transformed data. Values are shown as geometric means with 95% confidence intervals. *P < 0.05 compared with untreated BTBRob/ob and ^####^ *P* < 0.0001 compared with BTBR lean. Ena, enalapril.

Creatinine clearance (estimated GFR) was analyzed at 24 weeks of age. Creatinine clearance was increased in female BTBRob/ob mice, suggesting the presence of glomerular hyperfiltration (Fig. 2A). This prompted us to measure GFR by FITC‐sinistrin clearance in a small subset of age‐matched male BTBRob/ob mice. FITC‐sinistrin clearance was increased in the BTBRob/ob mice compared with lean healthy controls (Fig. 2B), confirming the presence of hyperfiltration in this model, which has not been reported before. Albumin excretion rate (AER) was analyzed at 24 weeks of age using 13‐h urine collections. Urinary albumin excretion rate was variable, but increased 13‐fold in BTBRob/ob mice compared with lean controls; however, AZD6610 or enalapril treatment had no statistically significant effect on albumin excretion (Fig. 3). Spot urine albumin:creatinine ratio (ACR) was also measured before the start of treatment and on two repeated occasions during treatment. Again, there was considerable variability in spot urine ACR, but on this measurement enalapril did reduce albuminuria (Fig. 4). Lean BTBR controls had lower urine ACR at all time points compared with BTBRob/ob controls.

### Structural alterations and podocyte markers

**Figure 5:**
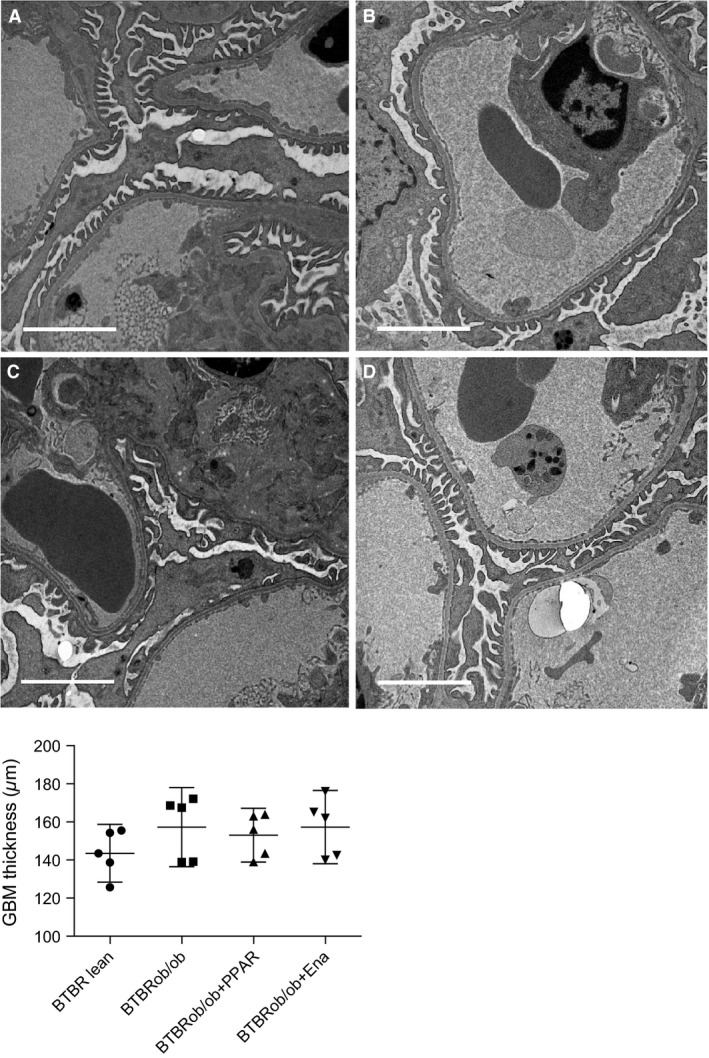
Glomerular basement membrane (GBM) thickness was unaltered at 24 weeks of age in the BTBRob/ob mouse. Glomerular basement membrane (GBM) thickness as measured by electron microscopy (EM) in five mice per group (females). One‐way ANOVA followed by Dunnett's multiple comparisons test were used and values are shown as means with 95% confidence intervals. A representative EM image of a capillary loop is shown for each treatment group. The scale bar in each image measures 3 *μ*m. (A) BTBR lean; (B) BTBRob/ob; (C) BTBRob/ob with PPAR *α*/*γ* agonist treatment; (D) BTBRob/ob with enalapril treatment. Ena, enalapril.

At 24 weeks of age, there were no apparent differences in GBM thickness in BTBRob/ob mice compared with lean controls and no obvious treatment effects could be detected (Fig. 5).

**Figure 6:**
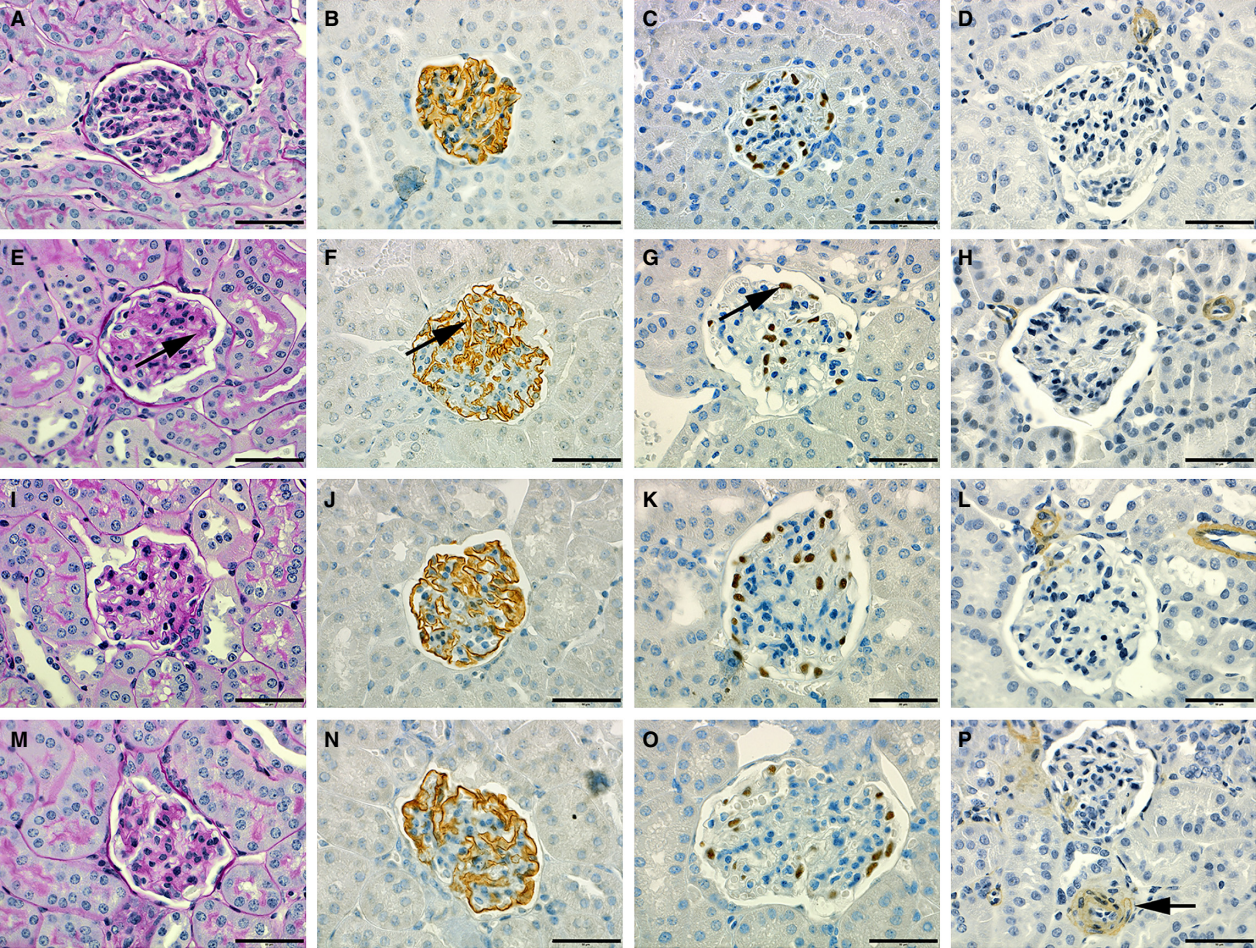
Mesangial matrix in BTBRob/ob was reduced by PPAR *α*/*γ* agonist treatment and ACE inhibition caused thickening of arteriolar vessel walls. Representative sections of female BTBR lean controls (A–D), BTBRob/ob (E–H), BTBRob/ob with PPAR *α*/*γ* agonist treatment (I–L), and BTBRob/ob with enalapril treatment (M–P). Sections were stained with PAS (A, E, I, and M), showing mesangial matrix expansion (arrow, E) and for podocyte markers nephrin (B, F, J and N; arrow F) and WT1 (C, G, K and O; arrow G) as well as for *α*‐SMA (D, H, L and P) showing arteriolar wall thickening (arrow P). The scale bar in each image measures 50 *μ*m. *α*‐SMA,* α*‐smooth muscle actin.

Mesangial matrix expansion was evident in all BTBRob/ob kidneys compared with lean controls and was reduced in AZD6610‐treated mice using semiquantitative scoring of PAS staining (average scores: 0.00 in lean controls; 2.58 in BTBRob/ob; 2.00 in BTBRob/ob + PPAR; 2.71 in BTBRob/ob + enalapril). Using semiquantitative scoring, the change (loss) in immunopositive staining for nephrin was found to be increased in all BTBRob/ob mice compared with lean mice (average score: 0.00 in lean controls; 2.67 in BTBRob/ob; 2.75 in BTBRob/ob + PPAR; 2.43 in BTBRob/ob + enalapril). The count of WT1‐positive cells per glomeruli was lower in all BTBRob/ob mice compared with lean BTBR mice (average count/glomerular area: 12.10 in lean controls; 9.50 in BTBRob/ob; 10.70 in BTBRob/ob + PPAR; 9.62 in BTBRob/ob + enalapril). Representative images of histopathology are shown in Figure 6.

**Figure 7:**
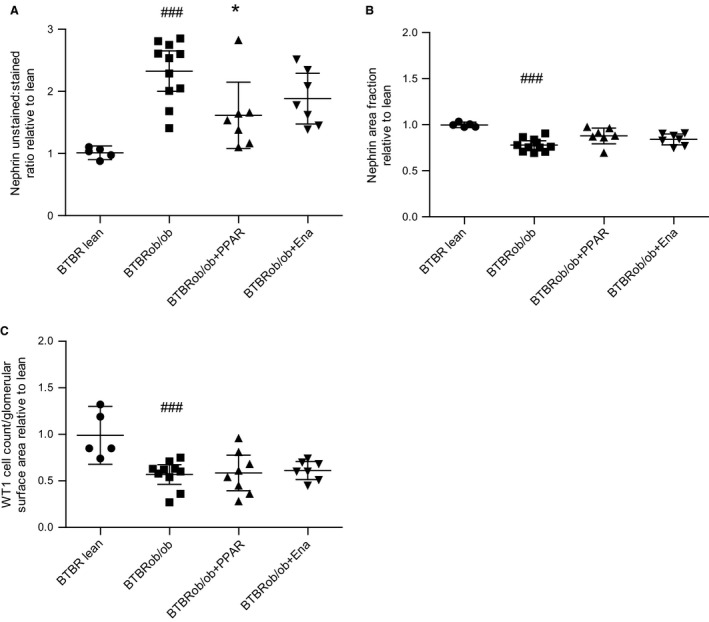
A novel automated image analysis method was used to quantify matrix accumulation and podocyte markers. (A) Relative ratios between unstained areas and nephrin‐stained areas (increased values indicate more damage). (B) Relative nephrin area fractions. (C) Relative WT1 cell counts/glomerular surface area. All data were divided by the geometric mean for the BTBR lean group and therefore represents fold change from this value. Data were log‐transformed prior to using one‐way ANOVA and Dunnett's post hoc test. *P < 0.05 and **P < 0.01 versus untreated BTBRob/ob. All figures show geometric means for each treatment group with 95% confidence intervals. Ena, enalapril.

Using automated image analysis, we quantified the ratio between the nephrin‐positive and ‐negative tissue areas in each individual glomerulus as an indirect measure of matrix accumulation. The data are shown as fold change from the lean BTBR group. There was a 2.3‐fold increase in matrix accumulation in BTBRob/ob mice, whereas AZD6610 reduced it to 1.6‐fold with a minimal, but not statistically detectable, difference in the enalapril group (1.9‐fold) compared with lean BTBR mice (Fig. 7A). There was one outlier in the BTBRob/ob + PPAR group (2.8‐fold increase) and this was the nonresponder described previously for its failure to show lowered glucose or triglycerides.

**Figure 8:**
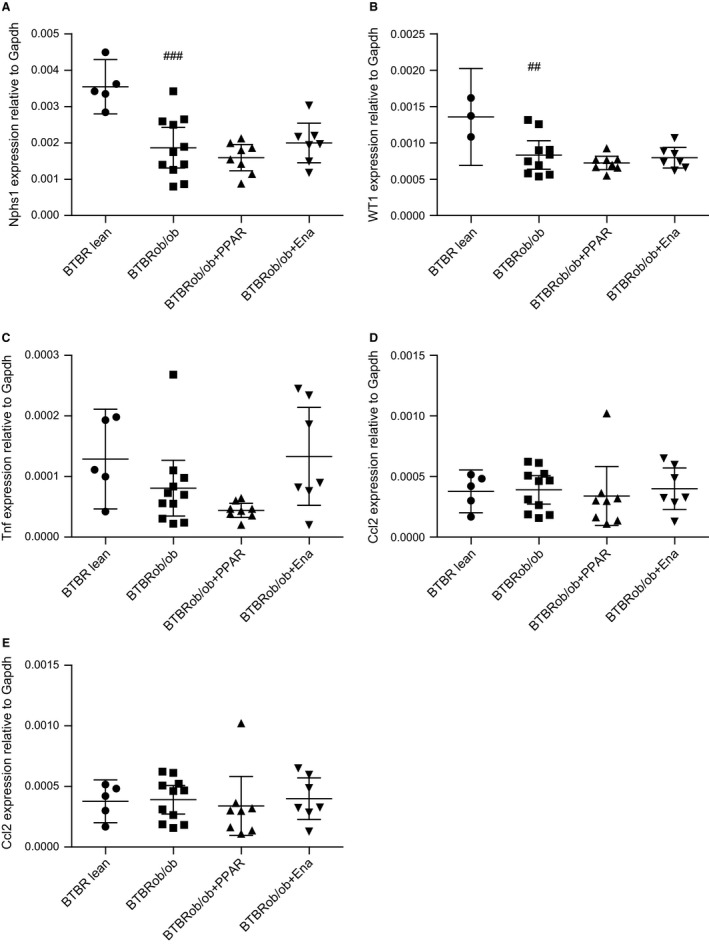
Gene expression of podocyte markers are decreased, whereas inflammatory markers are unchanged in BTBRob/ob female mice compared with lean controls. Levels of mRNA for podocyte markers nephrin (A) and WT1 (B), and inflammatory markers Tnf (C), Ccl2 (D), and CD68 (E) in renal cortex tissue at 24 weeks of age from lean BTBR (*n* = 5), BTBRob/ob controls (*n* = 12), and BTBRob/ob mice with AZD6610 (*n* = 8) or enalapril (*n* = 7) treatment. One‐way ANOVA followed by Dunnett's multiple comparisons test were used and data are shown as means with 95% confidence intervals. ^##^ *P* < 0.01 and ^###^ *P* < 0.001 compared with BTBR lean. Ena, enalapril; Nphs1, nephrin; Tnf, tumor necrosis factor; Ccl2, chemokine (C‐C motif) ligand 2, also referred to as monocyte chemoattractant protein 1 (MCP‐1).

Automated image analysis was then used to quantify nephrin and WT1 staining per glomerular area with the lean BTBR controls given the geometric mean of 1. There was decreased nephrin and WT1 positivity in all BTBRob/ob mice compared with lean BTBR mice (Fig. 7B–C). We could not detect any treatment effects on nephrin or WT‐1 levels in the groups we studied. The changes in podocyte markers in the BTBRob/ob compared with BTBR lean mice were also confirmed by gene expression analysis in renal cortical tissue (Fig. 8). The statistically significant difference in nephrin mRNA expression between BTBR lean and BTBRob/ob was reproducible when using two different housekeeping genes with a *P*‐value of 0.001 for Gapdh and 0.04 for Hprt.

An unexpected finding in this study was the presence of concentric minimal to moderate thickening of the vessel walls of the juxtaglomerular arterioles in all BTBRob/ob mice treated with enalapril. These findings were limited to the enalapril group (with one exception of a minimal finding) and the cellular wall thickening observed was positive for *α*‐smooth muscle actin (*α*‐SMA) staining (average score: 0.00 in lean controls; 0.00 in BTBRob/ob; 0.13 in BTBRob/ob + PPAR; 1.86 in BTBRob/ob + enalapril) (Fig. 6P). No *α*‐SMA positivity was observed in the tubulointerstitium.

Expression levels of the inflammatory markers TNF*α* (Tnf), MCP‐1(Ccl2), and CD68 were analyzed and we could not detect any differences between BTBRob/ob and BTBR lean controls or in response to the treatments used (Fig. 8C–E). Gene expression was normalized to both Gapdh and Hprt with the similar results (data only shown for Gapdh).

## Discussion

The BTBRob/ob mouse model is a relatively new model of DN that is thought to better reflect many of the typical features of human DN, including glomerular hypertrophy, mesangial matrix expansion, mesangiolysis, GBM thickening, podocyte loss, and albuminuria. In the present study, we were able to confirm most of these findings, including significant albuminuria, renal hypertrophy, mesangial matrix expansion, and reduced expression of podocyte markers consistent with cell injury and loss. However, in contrast to the study by Hudkins et al. (2010), we found more variability in some of the nephropathic features described, specifically GBM thickness, which could explain that we were unable to detect a difference between diabetic animals and lean controls. In addition, we assessed GFR for the first time in this model by measuring both creatinine and FITC‐sinistrin clearance. We found evidence for glomerular hyperfiltration in the BTBRob/ob mouse model at 24 weeks of age, which is a recognized feature of early DN, as found in most other diabetic mouse models, and may limit its use for translational studies of late stage human disease, and the interpretation (and even concern) over therapies that may also reduce GFR. Moreover, this mouse model does not exhibit hypertension (Hudkins et al. 2010). Hypertension is almost invariably present in human type 2 diabetes with nephropathy, and there is still a need for animal models that more closely resemble late human DN. Interventions that have become standard clinical practice in treating DN, and which have been shown to help slow progression, are antihypertensives, especially inhibitors of the renin–angiotensin–aldosterone system, and tighter glycemic control (Van Buren and Toto 2013). Yet despite these mainstays of current treatment, the number of patients progressing to end‐stage renal disease remains high. Leptin replacement in the BTBRob/ob mouse has been shown to reverse renal injury (Pichaiwong et al. 2013), but whether the beneficial effect of leptin is a result of improved metabolic control or of leptin signaling itself as a key determinant in the pathogenesis of diabetic nephropathy is unknown. It is known that glucose lowering per se may not improve renal function (Gangadharan Komala et al. 2014), at least in the short term. Other studies have shown that SGLT2 inhibition, which causes glycosuria and a lowered blood glucose concentration, can reduce renal inflammation, albuminuria, and mesangial expansion (Gembardt et al. 2014). PPAR agonists correct several metabolic derangements in diabetes, but also have anti‐inflammatory and antiatherogenic effects, as well as an action to lower blood pressure. Their potential renoprotective effects have been demonstrated in several studies of nondiabetic and diabetic kidney disease, with antifibrotic and podocyte protective effects described previously (Guan and Breyer 2001; Zuo et al. 2012).

In the current study, a slight reduction in PAS positivity was observed following AZD6610 treatment compared with BTBRob/ob controls, which was confirmed by both standard histopathological semiquantitative scoring and automated image analysis using nephrin‐negative staining as an indirect measure of glomerular matrix accumulation. These findings are consistent with previous studies (Park et al. 2006; Cha et al. 2007; Chodavarapu et al. 2013) of PPAR agonism in mouse models of type 2 diabetes. The novel and unbiased image analysis method used confirms the increase in PAS positivity in the BTBRob/ob mouse compared with lean controls, as well as the decrease following AZD6610 treatment. However, in contrast to the present findings, a recent study of the PPAR*α* agonist CP‐900691 in BTBRob/ob mice showed no effect on mesangial expansion (Askari et al. 2014). This discrepancy could be due to the use of a PPAR*α* versus a dual PPAR*α*/*γ* agonist (as in the present study), and/or early treatment as prevention, rather than later intervention, as well as duration of treatment.

The lipid‐lowering and insulin‐sensitizing effects of dual PPAR agonists are well‐known and have been reported in rodents and humans. In the present study, glucose and triglyceride levels were reduced; however, no change in insulin levels were apparent. PPAR*α*/*γ* agonism has been reported to restore adiponectin levels and to lower plasma insulin (Cha et al. 2007), where adiponectin is known to increase insulin sensitivity (Yamauchi et al. 2001; Kubota et al. 2002). Furthermore, podocyte foot process effacement and albuminuria have been observed in adiponectin knockout mice, while adiponectin administration has been shown to reverse these abnormalities (Sharma et al. 2008). Adiponectin treatment alone has been shown to reduce albuminuria and renal fibrosis, which are thought to result from the anti‐inflammatory and angiotensin‐blocking effects of adiponectin (Guo et al. 2014). Hyperinsulinemia is known to correlate with albuminuria (Sarafidis et al. 2010), but in the present study there were no detectable changes in insulin or adiponectin plasma concentrations in response to AZD6610 treatment. The lack of effect of AZD6610 on albuminuria could be due to a failure to restore adiponectin or lower insulin levels. Indeed, treatment with PPAR agonists has been shown to be more efficacious in correcting metabolic dysfunction and development of proteinuria when given as preventive treatment, rather than as an intervention, in aged animals with severe diabetes (Smith et al. 2000).

To explore the lack of effect of AZD6610 on albuminuria, the expression of the podocyte markers nephrin and WT1 was investigated. A lower count in podocyte cell number has been reported in the BTBRob/ob mouse model as early as 8 weeks of age (Hudkins et al. 2010), and although podocyte cell counting was not performed, nephrin and WT1 staining were found to be reduced compared with BTBR lean controls, both at the mRNA and protein expression levels at 24 weeks age. AZD6610 did not seem to improve any of the podocyte markers measured semiquantitatively. Podocyte protective effects and improved renal function have been reported with PPAR agonism in a nondiabetic model of podocyte injury (FSGS) (Yang et al. 2006), but in the current study, insufficient podocyte protection may partly explain the lack of effect on albumin excretion.

In previous studies with enalapril, there has been a robust decrease in albuminuria in BTBRob/ob mice (A. Ericsson, unpubl. obs.) and in the present study, albuminuria was decreased by enalapril treatment, although nephrin mRNA and protein expression appeared to be unchanged by enalapril. While no effect on matrix expansion was detectable in the present study, a modest effect has been reported previously in the BTBRob/ob mouse model (Pichaiwong et al. 2013), and may suggest some limitation to the benefits of RAS blockade in DN. There was also a trend for lower glucose concentrations in enalapril‐treated mice, without observable differences in daily food intake (data not shown) or body weight. Although glucose lowering in response to enalapril has not been reported in the BTBRob/ob model before, a reduced incidence in the onset of diabetes has been observed in clinical trials using ACE inhibitors (Solski and Longyhore 2008), and RAS blockade in a diabetic rat model has been shown to attenuate islet cell damage and to augment beta‐cell mass (Tikellis et al. 2004).

Inflammation is a recognized component of human and experimental DN and is typically characterized by infiltrating macrophages. There is some evidence for inflammation in the BTBRob/ob mouse model shown previously by Hudkins et al. (2010), who reported increased Mac‐2‐positive monocyte/macrophages in glomeruli of 22‐week‐old female mice. Treatment with enalapril has been shown to reduce the number of Mac‐2‐positive cells (Pichaiwong et al. 2013); however, the use of CP‐900691, a PPAR*α* agonist, did not affect Mac‐2‐positive cell numbers (Askari et al. 2014). In the current study, no statistically detectable change in mRNA expression of the inflammatory markers TNF*α* (Tnf), MCP‐1(Ccl2), or CD68 was observed in cortical tissue comparing BTBRob/ob with lean controls or between nontreated BTBRob/ob and enalapril or AZD6610 treated mice. The BTBRob/ob mouse model recapitulates more reliably glomerular changes seen in human DN and therefore gene expression analysis performed in isolated glomeruli may capture more readily any small inflammatory changes.

The finding of arteriolar remodeling was limited to the enalapril‐treated group and is similar to the feature of concentric hypertrophy seen in malignant hypertension. This is a novel and unexpected finding occurring in the absence of an elevated blood pressure in this model (Hudkins et al. 2010). This type of lesion has not been reported before, but it has been noted consistently in several previous unpublished studies (A. Ericsson, unpubl. obs.). The underlying mechanism for this is unclear, but since this form of vascular remodeling has not been reported in association with an ACE inhibitor or enalapril itself in patients, or in wild‐type rodent species, it may depend on the BTBR background or in deranged leptin functionality.

## Conclusions

Taken together, these results confirm previous findings of mesangial expansion, progressive albuminuria, and podocyte loss in the BTBRob/ob mouse. However, this model does not seem to recapitulate all the features of human DN, including robust GBM thickening, but it does show hyperfiltration that persists in older animals. Both PPAR*α*/*γ* agonism by AZD6610 treatment and ACE inhibition by enalapril treatment had no or only a limited effect on mesangial expansion and albuminuria. The vascular remodeling seen with enalapril treatment may limit its potentially beneficial effects in this particular animal model of DN.

## Conflict of Interest

The authors declare that there is no conflict of interest regarding the publication of this article. The study was funded by and carried out at AstraZeneca.
